# Design of Piezoelectric Ultrasonic Transducer Based on Doped PDMS

**DOI:** 10.3390/s21093123

**Published:** 2021-04-30

**Authors:** Ran Yang, Wenyi Liu, Wanjia Gao, Dingwei Kang

**Affiliations:** Key Laboratory of Instrumentation Science & Dynamic Measurement, Ministry of Education, North University of China, Taiyuan 030051, China; z523484935@163.com (R.Y.); 18810577682@163.com (W.G.); kangdingwei@petalmail.com (D.K.)

**Keywords:** 2-2 piezoelectric composites, PDMS, ultrasonic wave, ultrasonic transducer

## Abstract

The performance of the ultrasonic transducer will directly affect the accuracy of ultrasonic experimental measurement. Therefore, in order to meet the requirements of a wide band, a kind of annular 2-2-2 piezoelectric composite is proposed based on doped PDMS. In this paper, the transducer structure consisted of PZT-5A piezoelectric ceramics and PDMS doped with 3 wt.% Al_2_O_3_:SiO_2_ (1:6) powder, which constituted the piezoelectric composite. MATLAB and COMSOL software were used for simulation. Meanwhile, the electrode materials were selected. Then, the performance of the designed annular 2-2-2 ultrasonic transducer was tested. The simulation results show that when the polymer phase material of the piezoelectric ultrasonic transducer is doped PDMS, the piezoelectric phase and the ceramic substrate account for 70% of the total volume, the polymer phase accounts for 30% of the total volume, and the maximum frequency band width can reach 90 kHz. The experimental results show that the maximum bandwidth of −3 dB can reach 104 kHz when the frequency is 160 kHz. The results of the electrode test show that the use of Cu/Ti electrode improves the electrical conductivity of the single electrode. In this paper, the annular 2-2-2 transducer designed in the case of small volume had the characteristics of a wide frequency band, which was conducive to the miniaturization and integration of the transducer. Therefore, we believe that the annular 2-2-2 piezoelectric composite has broad application prospects.

## 1. Introduction

Ultrasound detection technology has been widely used in the fields of biomedicine [1,2], underwater detection [3], and aerospace [4]. Among them, the performance of piezoelectric ultrasonic transducers directly affects the measurement accuracy of the ultrasound detection technology. However, piezoelectric ultrasonic transducers are normally based on geometrical resonances of a piezoelectric element; this makes them present a reduced frequency bandwidth response compared to other non-resonant ultrasonic transducers (e.g., electrostatic transducers [5], etc.). Wide frequency band response is a key element in many different applications, such as, for example, ultrasonic communications and spectral materials characterization [6,7].

For this reason, in the past few decades, researchers have made some attempts to improve the performance of piezoelectric transducers. One way is to change the connection mode of the two-phase composite material. Among them, the 1-3 series and 2-2 series piezoelectric composite materials are representative. The cylindrical piezoelectric composite material designed by Li et al. [8] based on 1-3 PZT-5A/epoxy resin has a bandwidth of 7 kHz at a frequency of 72 kHz. According to Zhong et al. [9], based on the matching layer and 1-3 type PZT4/epoxy/silicone rubber, the cylindrical piezoelectric composite material has a bandwidth of 56 kHz. Qin et al. [10] reported that, based on the 1-3-2 type PZT-5H/epoxy resin designed cylindrical piezoelectric composite material, the bandwidth is 43.13 kHz. Hong-Wei et al. [11] demonstrated that based on the 2-2 type PZT-4/epoxy resin designed annular piezoelectric composite material, the bandwidth at 410 kHz frequency is 60 kHz. Lv et al. [12] showed that the matching layer and 1-3 type PZT-4/epoxy resin designed arc-shaped piezoelectric composite material has a bandwidth of 56.5 kHz. Liu et al. [13], based on the annular piezoelectric composite material designed by high frequency PMN-PT/epoxy 1–3, adjusted the shape and layout of the piezoelectric columns and arranged them in a annular array with a bandwidth of 90% at a frequency of 50 MHz. Based on the above references and references [14,15,16], it is shown that the annular structure plays an active role in expanding the bandwidth.

Another way to improve the performance of the piezoelectric transducer is to change the material of the polymer phase. Zhong et al. [9] demonstrated that the bandwidth of the cylindrical piezoelectric composite material designed based on the polymer phase material of epoxy resin/silicone rubber is 56 KHz; Rittenmyer et al. [17] designed a piezoelectric composite material containing 50% PZT and 50% silicone rubber. Sharma et al. [18] designed a composite material based on PZT-PDMS to improve vibration flexibility and auxiliary damping. At the same time, PDMS is widely used in acoustics. Research by Orbay et al. [19] showed that PDMS can be used as a channel in acoustic micro-mixers. Research by Yeo et al. [20] showed that PDMS can be used in the propagation of surface acoustic waves. A study by Acquaticci et al. [21] showed that PDMS can be done on axicon lens for focusing ultrasonic brain stimulation techniques. Research by Romo-Uribe et al. [22] showed that adding 10 wt.% of PDMS to epoxy resin can effectively improve the thermomechanical properties and toughness of epoxy resin. Compared with epoxy resin, PDMS has a lower acoustic impedance. Therefore, the acoustic impedance matching between the transducer and the air is improved to obtain a larger bandwidth. However, PDMS is also a high-attenuation acoustic material.

In order to improve the high attenuation of PDMS, fillers are doped in PDMS. The study by Wang et al. [23] showed that the addition of metal aluminum with a smaller Young’s modulus can obtain a larger electromechanical coupling coefficient. Sundar et al.’s [24] research indicated that the addition of Al particles improved dielectric and piezoelectric properties. Studies by Serbescu et al. [25] showed that silica particles are largely used as a reinforcing agent for silicone elastomers. Research by Kuryaeva and Hsu et al. [26,27] showed that Al2O3 and SiO2 can be used together in a ratio of 1:6.

To sum up, this paper proposes a miniature annular 2-2-2 piezoelectric composite based on doped PDMS. We deduced the equivalent parameter formula and selected the piezoelectric phase and polymer phase materials. Meanwhile, we used MATLAB and COMSOL Multiphysics software for simulation. In terms of the manufacturing process, the magnetron sputtering method was used to sputter the electrode, and the electrode material was selected. In this study, we compared the performance advantages and disadvantages of the transducer polymer phase material doped PDMS and traditional epoxy resin, and the material properties of the polymer phase of annular piezoelectric composite were optimized. In addition, compared to the original piezoelectric composite, the frequency band was broadened. At the same time, more possibilities are provided for the further study of piezoelectric composites.

## 2. Theoretical Research and Transducer Design

### 2.1. Derivation of Equivalent Parameters of Annular Piezoelectric Composite

**Figure 1:**
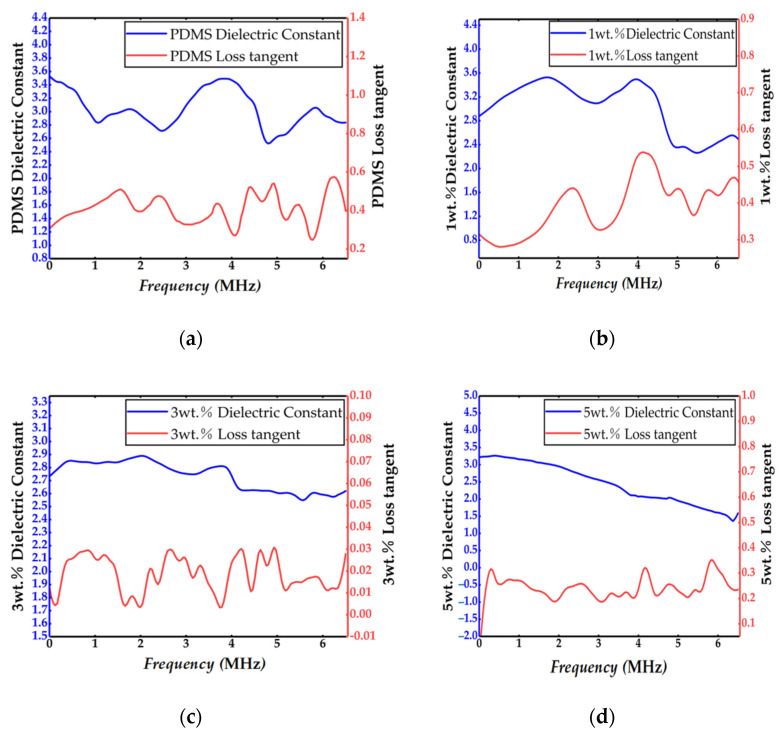
Comparison of dielectric constant and loss tangent of different doping volume ratios: (**a**) PDMS, (**b**) 1 wt.% Al_2_O_3_:SiO_2_ (1:6) powder, (**c**) 3 wt.% Al_2_O_3_:SiO_2_ (1:6) powder, and (**d**) 5 wt.% Al_2_O_3_:SiO_2_ (1:6) powder.

As shown in Figure 1, the Z direction is taken as the polarization direction, the piezoelectric phase is a transversely isotropic body, the polymer is an isotropic body, and the polymer has no piezoelectric effect.

The second type of piezoelectric equation, whose boundary conditions are mechanical clamping (S=0, T ≠ 0) and electrical short circuit (E=0, D ≠ 0), is:

(1) $$\left\{ \begin{array}{l} D=\varepsilon^{S}E+eS\\ T=-e^{t}E+c^{E}S \end{array}\right.,$$

The *Z* direction is set as the polarization direction, so when the 2-2 piezoelectric composite material is stretched along the polarization direction, it will not cause the coupling effect of shear deformation. Therefore, $T_{4}{=T}_{5}{=T}_{6\;}{=0,\;E}_{1}{=E}_{2}=0$ is used to process the equation.

According to Newnham’s series and parallel model, Smith’s model, and Khan’s derivation theory [28,29,30,31,32,33], the stress and strain relations along the *X*, *Y*, and *Z* directions are obtained. In the formula, $V_{c}$ represents the piezoelectric phase of 2-2 type piezoelectric composites in the overall volume percentage, the right superscript *C* represents the piezoelectric phase, the right superscript *P* represents the polymer phase, and the right superscript *CP* represents the 2-2 type piezoelectric composites as a whole.

In the *X* direction, the piezoelectric phase and the polymer are connected in series, each stress is equal, and the strain is the sum of each corresponding variation:

(2) $$T_{1}{}^{cp}=T_{1}{}^{c}=T_{1}{}^{p},$$

(3) $$S_{1}{}^{cp}=V_{c}\times S_{1}{}^{c}+(1-V_{c})\times S_{1}{}^{p},$$

In the *Y* direction, the piezoelectric phase and the polymer are connected in series, each stress is equal, and the strain is the sum of each corresponding variation:

(4) $$T_{2}{}^{cp}=T_{2}{}^{c}=T_{2}{}^{p},$$

(5) $$S_{2}{}^{cp}=V_{c}\times S_{2}{}^{c}+(1-V_{c})\times S_{2}{}^{p},$$

For the *Z* direction, the piezoelectric phase is parallel to the polymer, the strain is equal, the stress is the sum of the corresponding forces, the electric field intensity is equal, and the electric displacement component is the sum of the electric displacement components of each phase.

(6) $$S_{3}{}^{cp}=S_{3}{}^{c}=S_{3}{}^{p},$$

(7) $$T_{3}{}^{cp}=V_{c}\times T_{3}{}^{c}+(1-V_{c})\times T_{3}{}^{p},$$

(8) $$E_{3}{}^{cp}=E_{3}{}^{c}=E_{3}{}^{p},$$

(9) $$D_{3}^{cp}=V_{c}\times D_{3}^{c}+(1-V_{c})\times D_{3}^{p},$$

After equivalent transformation, the equivalent equation with $S_{1}{,\;S}_{2}{,\;S}_{3}{,\;D}_{3}$ as independent variables is obtained. Compared with the constitutive equation, the theoretical calculation formula of macroscopic equivalent property parameters of 2-2 piezoelectric composites is obtained.

(10) $$C_{11}^{Ecp}+C_{12}^{Ecp}=V_{c}(\frac{1-C_{12}^{c}}{{\left(C_{11}^{c}\right)}^{2}-{\left(C_{12}^{c}\right)}^{2}})+(1-V_{c})(\frac{C_{11}^{p}-C_{12}^{p}}{{\left(C_{11}^{p}\right)}^{2}-{\left(C_{12}^{p}\right)}^{2}}),$$

(11) $$m=C_{11}^{Ecp}+C_{12}^{Ecp},$$

(12) $$n=\left(\frac{V_{c}C_{13}^{c}}{C_{11}^{c}+C_{12}^{c}}+\frac{(1-V_{c})C_{12}^{p}}{C_{11}^{p}+C_{12}^{p}}\right),$$

(13) $$C_{13}^{Ecp}=mn,$$

(14) $$C_{33}^{Ecp}=V_{c}[C_{33}^{c}-\frac{2{\left(C_{33}^{c}\right)}^{2}}{C_{11}^{c}+C_{12}^{c}}]+(1-V_{c})\frac{C_{11}^{p}(C_{11}^{p}+C_{12}^{p})-2{\left(C_{12}^{p}\right)}^{2}}{C_{11}^{p}+C_{12}^{p}}+2mn^{2},$$

(15) $$e_{33}^{cp}=V_{c}[e_{33}^{c}-\frac{2e_{31}^{c}C_{13}^{c}}{C_{11}^{c}+C_{12}^{c}}]+\frac{2mnV_{c}e_{31}^{c}}{C_{11}^{c}+C_{12}^{c}},$$

In the same way, the piezoelectric equations of the first kind, whose boundary conditions are mechanical freedom *(*T=0, S ≠ 0*)* and electrical short circuit *(*E=0, D ≠ 0*)*, are applied. The equivalent properties of 2-2 piezoelectric composites can be calculated as follows:

(16) $$S_{33}^{Ecp}=\frac{S_{33}^{Ec}\times S_{33}^{Ep}}{V_{c}S_{33}^{Ep}+(1-V_{c})S_{33}^{Ec}},$$

(17) $$d_{33}^{cp}=\frac{V_{c}d_{33}^{c}S_{33}^{Ep}+(1-V_{c})d_{33}^{p}S_{33}^{Ec}}{V_{c}S_{33}^{Ep}+(1-V_{c})S_{33}^{Ec}},$$

(18) $$S_{13}^{Ecp}=\frac{V_{c}S_{13}^{Ec}S_{33}^{Ep}+(1-V_{c})S_{13}^{Ep}S_{33}^{Ec}}{V_{c}S_{33}^{Ep}+(1-V_{c})S_{33}^{Ec}},$$

### 2.2. Piezoelectric Phase Material Selection

In order to achieve the best electrical and acoustic properties of annular composites, the choice of materials is very important. PZT was selected as the piezoelectric phase and ceramic substrate. The common results are shown in Table 1. The data in the table are temperature (°C), piezoelectric constant (Pc/N), relative dielectric constant, loss angle tangent, electromechanical coupling coefficient, density (10^3^ Kg/m^3^), piezoelectric voltage constant, and sound velocity (m/s).

From the parameters in Table 1, PZT-4 had a low loss coefficient, low density, high electromechanical coupling coefficient, longitudinal piezoelectric strain constant, and Curie temperature, and could be applied to transmitting transducers. Although the dielectric constant of PZT-8 was low, the loss coefficient was the lowest, the tensile strength and stability were good, and it was more suitable for high-power transducers. PZT-5A had a high longitudinal piezoelectric strain constant; relative dielectric constant, density, and Curie temperature; and had good temperature stability. It was suitable for application in high temperature environment as a transmitter and receiver dual-purpose transducer. Therefore, PZT-5A was selected as the piezoelectric phase material.

### 2.3. Polymer Phase Material Selection

The piezoelectric ultrasonic transducer requires the wafer to have a larger electromechanical coupling coefficient and a wider bandwidth in order to obtain a higher conversion coefficient. However, the electromechanical coupling coefficient and bandwidth of PZT-5A are small, so the choice of polymer phase materials is particularly important. At present, the common material on the market that can be used for the polymer phase is epoxy resin. However, many experiments have shown that when the polymer phase is epoxy resin, the thickness electromechanical coupling coefficient is small, only about 0.6. At the same time, the bandwidth of piezoelectric ultrasonic transducers using epoxy resin as the polymer phase material is relatively small. In order to improve the performance of piezoelectric transducers, many scholars have changed the material of the polymer phase. Among them, the research of Sharma et al. [18] and Romo-Uribe et al. [22] showed that PDMS can improve the flexibility of vibration, auxiliary damping, and thermomechanical properties. This is because PDMS has a lower acoustic impedance than epoxy resin and can better match with air. At the same time, it is a commonly used flexible sensor material that has strong biochemical stability, high and low temperature stability, good biocompatibility, a relatively simple production method, and low cost, so the polydimethylsiloxane (PDMS) was planned to be selected as the polymer phase in this experiment.

**Figure 2:**
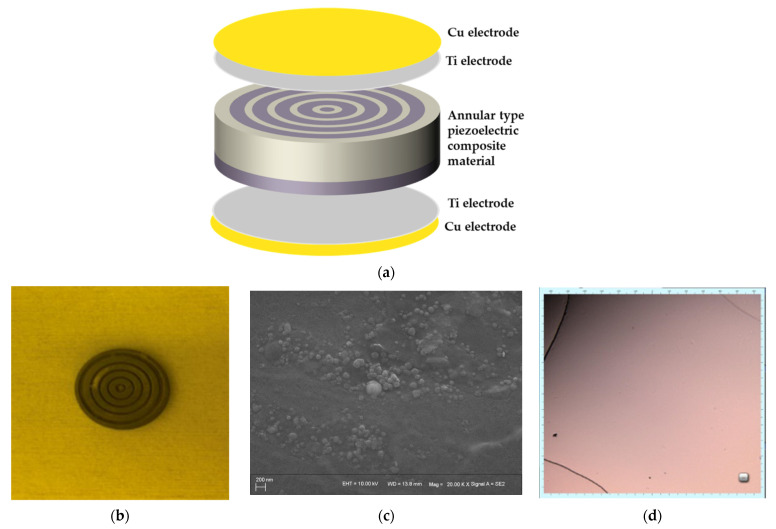
Structure diagram of annular type piezoelectric transducer: (**a**) theoretical structure of piezoelectric transducer, (**b**) PZT-5A after laser marking machine processing, (**c**) SEM image of doped composite material, and (**d**) surface condition of electrode under confocal microscope.

However, PDMS is also a high-attenuation acoustic material. In addition, according to Table 2 and referring to relevant information, the relative dielectric constant of polymer phase material PDMS was about 2.75, and the tangent of loss angle was about 0.375. The relative dielectric constant of PZT-5A was about 1700, and the positive angle tangent of loss was about 0.02.That is, filling the polymer phase would lead to an increase in the tangent of the loss angle. At the same time, the loss tangent value is an important basis for judging the performance of piezoelectric materials. The smaller the loss tangent value, the better the material performance and the smaller the power loss of the transducer itself. Due to the large difference of thermal expansion coefficient, the metal electrode deposited by sputtering directly on the electrode would be easy to crack, which would affect the conductive effect of the electrode. Therefore, in order to solve the abovementioned problems of PDMS, based on the research of many scholars [23,24,25,26,27], the PDMS was doped with Al_2_O_3_:SiO_2_ (1:6) powder, with the hope of reduce the attenuation, increasing the adhesion of the electrode, and reducing the loss tangent. Experiments have proved that when doped with 3 wt.% Al_2_O_3_:SiO_2_ (1:6) powder, the loss tangent of the polymer phase is about 0.01~0.03, and the adsorption force to the electrode is the best (see the following comparison). Figure 2 is a comparison of the loss tangent values of the polymer with different doping volume ratios tested by the vector network analyzer and the pure PDMS.

### 2.4. Design and Simulation of Transducer

#### 2.4.1. Annular Structure Design

The annular 2-2-2 piezoelectric composite is composed of type 2-2 piezoelectric composite and ceramic substrate in series. PZT-5A was chosen as the piezoelectric phase material, and PDMS doped with 3 wt.% Al2O3:SiO2 (1:6) [26,27] powder as the polymer phase material. The annular structure is shown in Figure 2.

#### 2.4.2. Numerical Calculation of Optimal Volume Percentage Range

**Figure 3:**
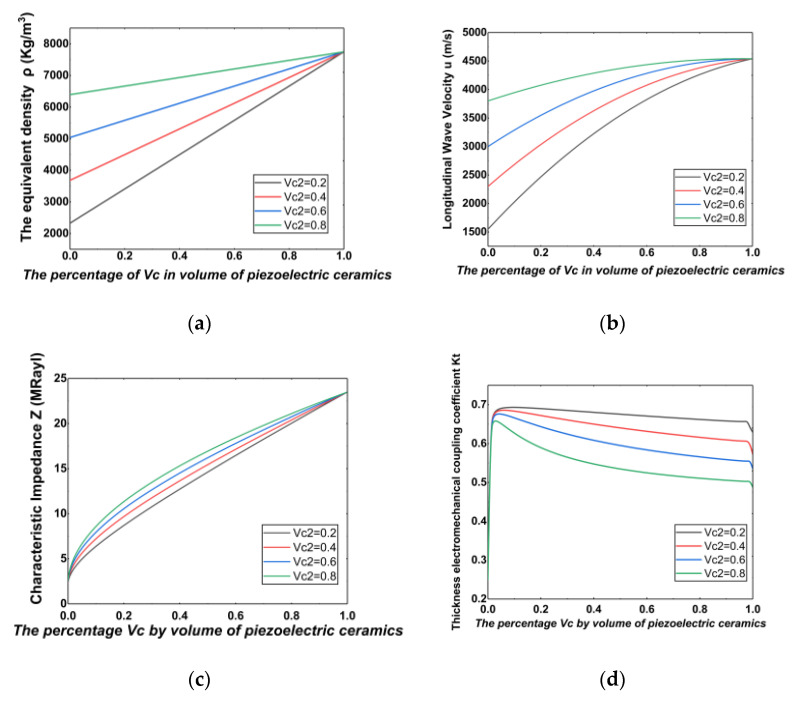
(**a**) The relation curve of equivalent density ρ with $V_{c}$ and $V_{c2}$, (**b**) the curve of longitudinal wave sound velocity μ as a function of $V_{c}$ and $V_{c2}$, (**c**) the variation curve of characteristic impedance Z with respect to $V_{c}$ and $V_{c2}$, and (**d**) the variation curve of thickness electromechanical coupling coefficient of thickness $k_{t}$ with respect to $V_{c}$ and $V_{c2}$.

We use MATLAB to calculate the equivalent performance parameters of piezoelectric composites. We preliminarily determined the range of the optimal volume percentage and obtained the curves of equivalent density ρ, electromechanical coupling coefficient of thickness $k_{t}$, longitudinal wave sound velocity μ, and characteristic impedance Z with the proportion of piezoelectric phase $V_{c}$ and ceramic substrate $V_{c2}$, respectively, as shown in Figure 3.

From Figure 3a we can see that the equivalent density ρ increased with the increase of $V_{c}$ and $V_{c2}$. It can be seen from (b) that the longitudinal wave sound velocity increased rapidly with the increase of $V_{c}$. When μ > 0.3, the growth rate gradually flattened out and finally reached a fixed value. It can be seen from Figure 3c that the characteristic impedance Z increased with the increase of $V_{c}$ and $V_{c2}$. We can see from Figure 3d that when $V_{c}$ was very small, the electromechanical coupling coefficient of thickness $k_{t}$ of the composite material was also very small. When $V_{c}$ increased between 0 and 0.1, $k_{t}$ also increased rapidly. When $V_{c}$ > 0.1, as $V_{c}$ and $V_{c2}$ increased, $k_{t}$ decreased slowly. As $V_{c}$ approached 1, $k_{t}$ decreased rapidly. It can also be seen from Figure 3d that for the annular fitting result, $k_{t}$ had a minimum of about 0.45 and a maximum of about 0.65. In general, $k_{t}$ was greater than that of the PZT-5A material itself ($k_{t}$ = 0.49). Therefore, the design of the experiment can obtain a higher thickness electromechanical coupling coefficient than the intrinsic electromechanical coupling coefficient of the material.

#### 2.4.3. Optimal Size Simulation

**Figure 4:**
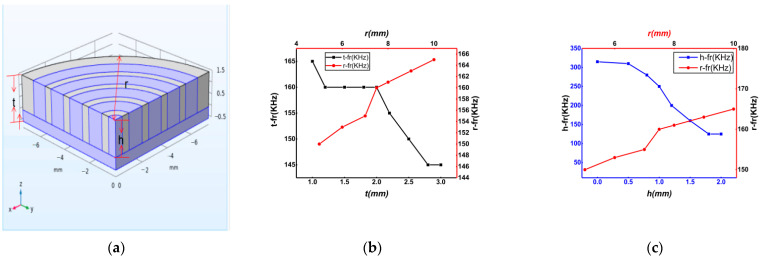
(**a**) Cross-section of the model, (**b**) fitting figure of thickness *t* and radius *r*, and (**c**) fitting figure of ring height *h* and radius *r*.

We used COMSOL Multiphysics software to simulate the structure of the annular 2-2-2 piezoelectric composite. Simultaneously, simulations were performed under different thicknesses *t* (mm), piezoelectric ring heights *h* (mm) and piezoelectric ceramic sheet diameters *r* (mm). The results are shown in Figure 4. The simulation results show that when the polymer phase material of the piezoelectric ultrasonic transducer was doped PDMS, the piezoelectric phase and the ceramic substrate accounted for 70% of the total volume, and the polymer phase accounted for 30% of the total volume, the performance of the transducer was the best.

## 3. Preparation Method of Transducer Substrate and Electrode

**Figure 5:**
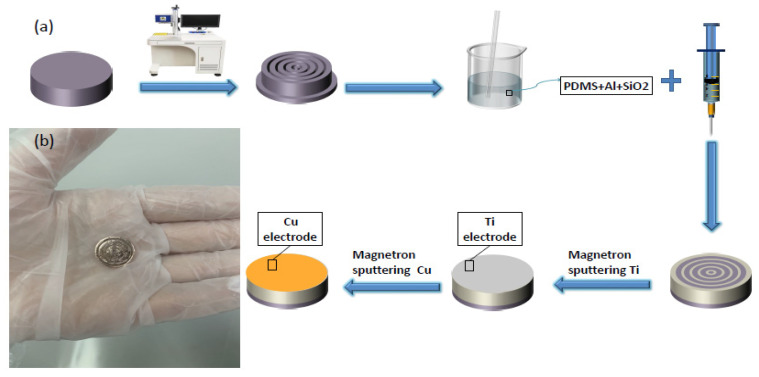
Flow chart and actual samples: (**a**) is the production flow chart of the annular piezoelectric composite material, and (**b**) is the actual production sample.

Figure 5 is the fabrication process of the annular 2-2-2 transducer. First of all, a laser marking machine was used to cut out the annular type structure, the composite material was put into acetone solvent by an ultrasonic shock cleaning machine, and the impurities produced by laser cutting were washed away. Then, nitrogen was used to blow dry and put in the working area of 100 levels of cleanliness for standby. After mixing the PDMS and the curing agent at a ratio of 10:1, it was stirred for 30 min and a vacuum machine was used for vacuum treatment. Then, the polymer was poured into the structural skeleton with a 0.3*4 mm syringe, and the filling height and the number of grams of polymer were controlled to ensure that the polymer phase was evenly poured. Finally, the composite material was placed on the drying table to cure the polymer. The volume percentage of piezoelectric phase can be controlled by controlling the power of laser marking machine, and the fabrication process is simple, the cost is very low, and it is very suitable for mass production.

Electrodes prepared by magnetron sputtering can be optimized by controlling the parameters of target material, composition ratio, sputtering power, and sputtering time. At the same time, the purity and consistency of the electrode obtained by the sputtering coating method are high. Therefore, this experiment used magnetron sputtering to prepare the electrodes. Before magnetron sputtering, the rougher annular 2-2-2 composite material obtained in the previous step was thinned and polished by a chemical mechanical thinning polishing machine. The thinned and polished material was then etched with oxygen ions in order to make the electrode adhere better to the material. Finally, the composite material was put into acetone or alcohol solvent by ultrasonic shock cleaning machine again to remove all kinds of impurities and surface stains. The composite was blow dried with nitrogen and then we waited for the sputtering electrode.

### 3.1. Simulation Results

**Figure 6:**
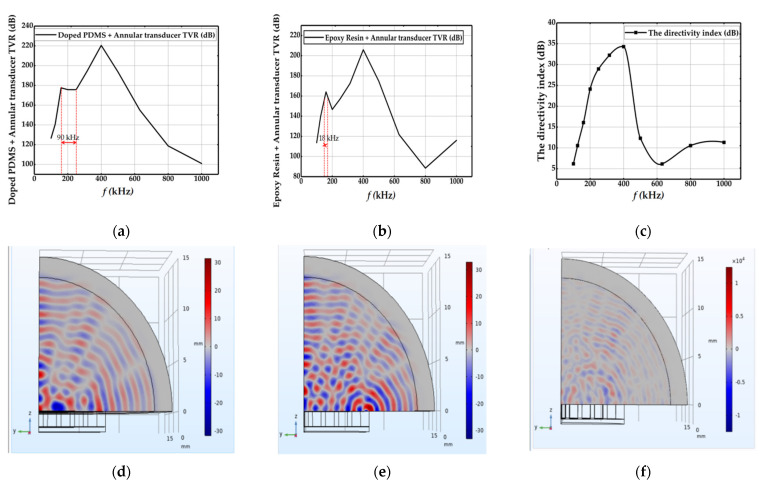
Simulation results of the annular transducer: (**a**) transmission voltage response of the polymer phase doped PDMS annular transducer, (**b**) transmission voltage response of the polymer phase epoxy resin annular transducer, (**c**) directivity index of the polymer phase doped PDMS annular transducer, (**d**) the total sound pressure field of 160 kHz of the polymer phase doped PDMS annular transducer, (**e**) the total sound pressure field of 2500 kHz of the polymer phase doped PDMS annular transducer, and (**f**) the total sound pressure field of 400 kHz of the polymer phase doped PDMS annular transducer.

We used COMSOL simulation software to simulate the vibration mode of the annular 2-2-2 transducer of PDMS doped with 3 wt.% Al2O3: SiO2 (1:6) powder in air. An infinite air domain was added to the annular transducer, a perfect matching layer (PML) was added to the outermost layer to simulate the absorption of sound waves in the process of propagation away from the sound source, and an outfield calculation was established at the interface boundary between the air domain and the PML domain. The transmission voltage response TVR and directivity index DI were calculated by Formulas (19) and (20), respectively. Among them, the transmission voltage response refers to the free field sound pressure generated by the transmission transducer in the specified direction, 1 m away from its effective sound center. In fact, the directivity index is the number of decibels higher the sound level on the directivity transmitting sound axis is than the sound level of the non-directivity transmitting sound field at the same distance. The simulation results are shown in Figure 6.

(19) $$TVR=S_{vL}=20log\left[\frac{S_{v}}{{\left(S_{v}\right)}_{0}}\right],$$

where ${{(S}_{v})}_{0}=1\mu \mathrm{Pa}/{V=10}^{6}\mathrm{Pa}/V$.

(20) $$DI=10log\frac{I_{D}}{I_{ND}},$$

In Formula (20), $I_{D}$ represents the sound intensity with directivity, and $I_{ND}$ represents the sound intensity without directivity.

The simulation results in Figure 6a show that the transmission voltage response (TVR) of the annular 2-2-2 sensor with polymer phase of PDMS doped with 3 wt.% Al_2_O_3_: SiO_2_ (1:6) powder varied with frequency hen the piezoelectric phase and the ceramic matrix accounted for 70% of the total volume and the polymer phase accounted for 30% of the total volume. It can be seen from the figure that when the frequency was 160 kHz, the bandwidth of the sensor −3 dB was about 90 kHz.

The simulation result of Figure 6b shows that when the polymer phase material was epoxy resin, the transmission voltage response (TVR) of the annular 2-2-2 sensor changed with frequency when the piezoelectric phase and the ceramic matrix accounted for 70% of the total volume and the polymer phase accounted for 30% of the total volume. It can be seen from the figure that when the frequency was 160 kHz, the bandwidth of the sensor −3 dB was about 18 kHz.

The reason for the difference is that the acoustic impedance of PDMS is lower than that of epoxy resin. Therefore, the acoustic impedance matching between the transducer and the air is improved to obtain a larger frequency band width.

We can see from Figure 6c the directivity index (DI) curve of the annular 2-2-2 transducer using COMSOL Multiphysics software. In the range of 160 kHz to 250 kHz, the directivity index of the transducer increased from 16 dB to 29 dB, whereas its transmission voltage response remained almost constant. Because of this feature, the application of the transducer in this working range is very wide.

Figure 6d–f show the distribution of the total sound pressure field simulated by the annular transducer at the frequencies of 160 kHz, 250 kHz, and 400 kHz, respectively. It can be seen from the figure that the sound pressure converged in red and blue. The more obvious the red and blue position in the picture, the stronger the sound field at that position. The lower the frequency of the sound wave, the longer its wavelength, and the more obvious the sound field distribution.

### 3.2. Testing of Electrodes

**Figure 7:**
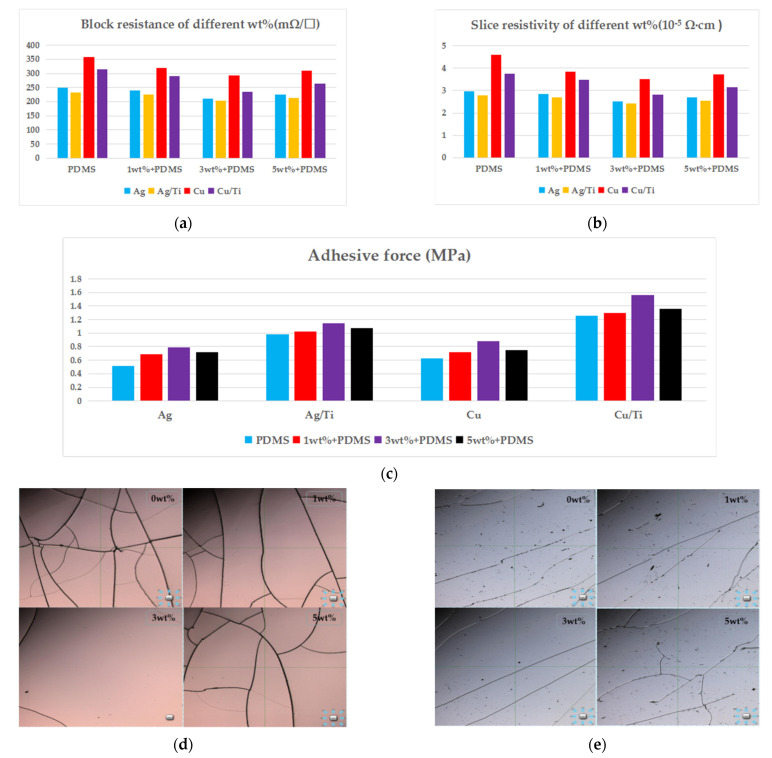
Test results of the electrode: (**a**) block resistance, (**b**) slice resistance, (**c**) adhesive force, (**d**) confocal microscope image of sputtered Cu/Ti electrode at different wt.%, and (**e**) confocal microscope image of sputtering Ag/Ti electrode at different wt.%.

Due to the different thermal expansion coefficient of different metal targets, in order to enhance the adhesion of the electrode, a four-probe tester was used. Under the condition of sputtering with the same thickness, samples with different wt.% were tested by sputtering with different metal targets; that is, the electrical conductivity of the electrodes sputtered by Ag,Cu and Ag/Ti,Cu/Ti was tested, where the transition layer was Ti. Six different points were selected for measurement, and then the test results of the same sputtered layer were averaged. The measured sheet resistivity and sheet resistance are shown in Figure 7a,b. As can be seen from the figure, in the case of the same sputtering thickness, the conductive performance of the electrode with the transition layer Ti was significantly better than that of the sputtering layer without the transition layer.

At the same time, the adhesion of the interface between the electrode and the composite material was tested by the direct pull method. The lead wire was soldered onto the electrode film with a diameter of about 2 mm and an area of about 3 mm square. The test results of the electrode are shown in the figure. It can be seen from the data in Figure 7c that Cu/Ti as the electrode had the best adhesion force.

Figure 7d,e show the electrode adhesion after sputtering Cu/Ti and Ag/Ti electrodes on the composite materials doped with different wt.% under confocal microscope. It can be clearly seen from Figure 7e that the electrode sputtered with Ag/Ti had cracks and obvious electrode pores, resulting in poor electrode continuity and affecting the conductive effect. Although there were cracks in the sputtered Cu/Ti electrode, there were no electrode pores. When doped with 3 wt.%, the crack of the electrode was obviously reduced, and the adhesion and conductivity were the best.

To sum up, we chose Cu/Ti as the electrode of annular transducer.

### 3.3. Performance Testing

The annular piezoelectric composite material was prepared by the method described in Figure 5. At the same time, ultrasonic precision impedance analyzer (E4990A, KEYSIGHT, Beijing, China) was used for testing. In the test process, the wire length between the test sample and the instrument should be reduced as much as possible so the effect of the equivalent resistance of the piezoelectric vibrator on the test results can be ignored, so that the measured minimum impedance frequency and maximum impedance frequency are approximately equal to the resonant frequency and anti-resonant frequency.

The following electrical parameters of the sample were tested: primary resonance frequency $f_{r}$ (kHz), secondary resonance frequency $f_{r2}$ (kHz), thickness electromechanical coupling coefficient $K_{T}$*,* longitudinal wave velocity *μ* (m/s), density *ρ* (Kg/m^3^), characteristic impedance *Z* (MRayl), and relative dielectric constant $\varepsilon_{r}$. The test results are shown in Table 3.

The test results were relatively close to the data calculated by the data analysis software and COMSOL Multiphysics software. The error between the experimental value and the theoretical value is shown in Table 4. Among them, $\Delta f_{r}\%\;\mathrm{and}\;\Delta f_{r2}$% are the percentages of the error values of primary resonance frequency and secondary resonance frequency, respectively, whereas $\Delta K_{T}$%, Δ*μ*%, Δ*ρ*%, and Δ*Z*% are the percentages of the error values of thickness electromechanical coupling coefficient, longitudinal wave sound velocity, equivalent density, and characteristic impedance, respectively. The calculation method of all error values was the ratio of the actual value and the theoretical value, and the results were positive and retained two decimal places. Therefore, it can be considered that the above theoretical parameter analysis and simulation results are close to the actual calculated values.

### 3.4. Bandwidth and Relative Pulse Echo Sensitivity Test

**Figure 8:**
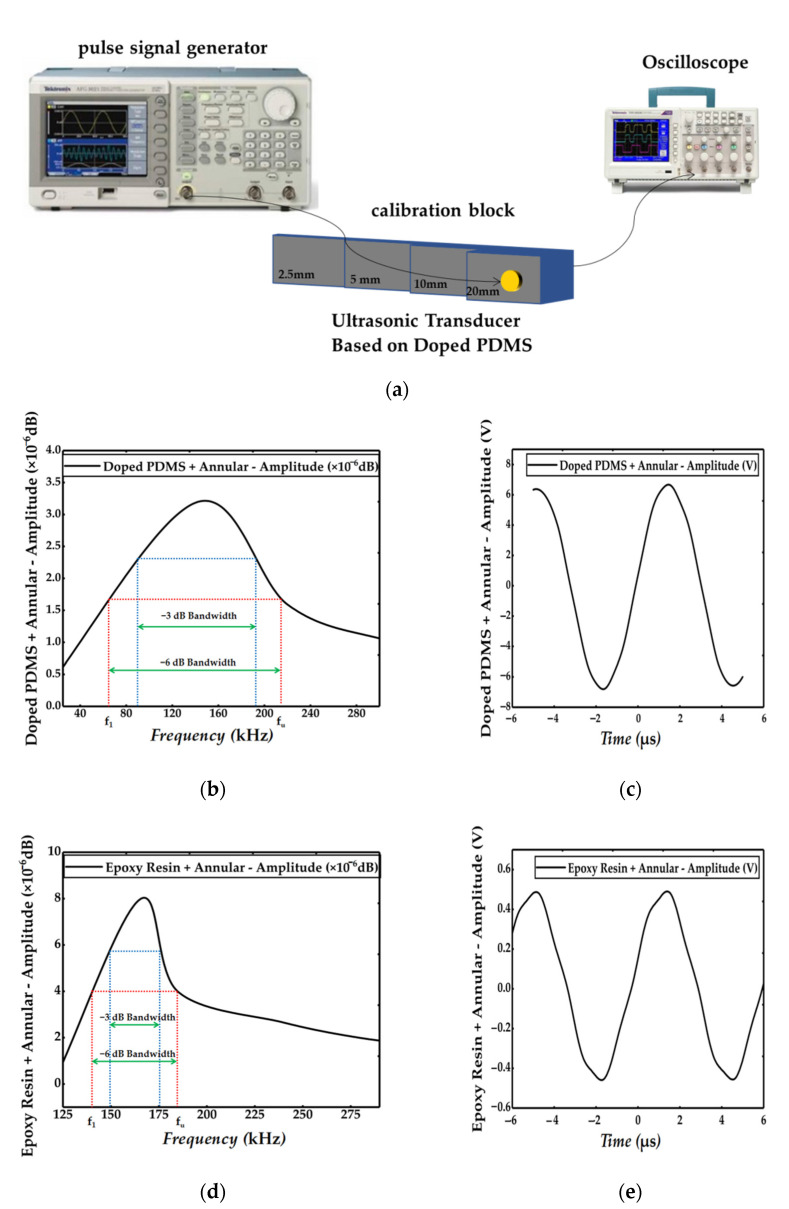
Bandwidth and relative pulse echo sensitivity test results: (**a**) designed diagram of experimental platform, (**b**) spectrum of pulse echo detection of the doped PDMS and annular, (**c**) time domain response of the doped PDMS and annular, (**d**) spectrum of pulse echo detection of the epoxy resin and annular, and (**e**) time domain response of the epoxy resin and annular.

The pulse echo method of ultrasonic nondestructive testing was used to test the bandwidth. The experiment needed a pulse signal generator (AFG3021C, Tektronix, Shanghai, China), an oscilloscope with FFT function (TDS 1001B, Tektronix, Shanghai, China), and four order probe calibration blocks with thicknesses of 2.5, 5, 10, and 20 mm. The length of the near-field region [21] was calculated with Formulas (21) and (22), and the width of the calibration block was larger than the diameter of the tested sample, so the part with a thickness of 20 mm was selected for testing. The design of the experimental platform is shown in Figure 8a. In order to avoid interference, a double probe detection method was used in the experiment; that is, a probe transmitted and a probe received, and a pulse signal generator was used to excite a square wave signal with 160 kHz frequency and 1V amplitude. For samples 1–5, the piezoelectric phase material was PZT-5A, the polymer phase material was PDMS doped with 3 wt.% Al2O3: SiO2 (1:6) powder, the piezoelectric phase and ceramic matrix accounted for 70% of the total volume, and the polymer phase accounted for 30% of the total volume. The experimental results of samples 1–5 are shown in Figure 8b,c and Table 5. For samples 6–10, the piezoelectric phase material was PZT-5A, the polymer phase material was epoxy resin, the piezoelectric phase and ceramic matrix accounted for 70% of the total volume, and the polymer phase accounted for 30% of the total volume. The experimental results of samples 6–10 are shown in Figure 8d,e and Table 6. The relative pulse echo sensitivity S_rel_ (dB) was calculated by Formula (23). The *f_u_* and *f*_1_ are the high and low frequencies at the echo amplitude 50% (−6 dB) lower than the maximum echo amplitude. V_P-P_ is the peak-to-peak voltage displayed by the oscilloscope from the sample to be measured.

(21) $$N=\frac{D^{2}}{4\lambda }=\frac{A}{\pi \lambda },$$

In Formula (21), *D* is the diameter of the transducer m, *A* is the effective area of the transducer m^2^, and *λ* is the wavelength of the ultrasonic wave propagating in the medium, which can be calculated using Formula (22):

(22) $$\lambda =\frac{c}{f},$$

where *c* is the wave speed of ultrasonic wave propagation in the medium m/s, and *f* is the frequency of ultrasonic wave Hz.

(23) $$S_{rel}=20*lg(U_{e}/U_{a}),$$

where *U_e_* and *U_a_* are the peak-to-peak voltage (V) of the return wave from the specified reflector and the peak-to-peak voltage (V) applied to the tested sample, respectively.

## 4. Conclusions

In summary, a piezoelectric ultrasonic transducer based on doped PDMS as a polymer phase material was designed. First, the formula for equivalent parameters was derived, and then the optimal size and simulation results were determined with MATLAB software and COMSOL Multiphysics software. For the polymer phase, PDMS was used to replace the common epoxy resin available in the market, and the original PDMS was doped with 3 wt.% Al_2_O_3_:SiO_2_ (1:6) powder, which broadened the frequency band. The experimental results show that the maximum bandwidth of −3 dB could reach 104 kHz when the frequency was 160 kHz. The results of the electrode test show that the use of Cu/Ti electrode improved the electrical conductivity of the single electrode. At the same time, due to the unique copolymerization and adhesion of PDMS, it could be flexibly doped with materials of different properties to change the performance of the polymer phase. Thus, it can be argued that the annular 2-2-2 piezoelectric composite has broad application prospects.

## Figures and Tables

**Table 1:** Common PZT performance parameters.

Materials	$T_{c}({}^{\circ}C)$	$d_{33}$	$\varepsilon_{33}/\varepsilon_{0}$	tanδ	$k_{t}$	ρ	$g_{33}$	C
PZT-4	328	289	1300	0.005	0.51	7.5	2.6	4000
PZT-5A	365	374	1700	0.02	0.49	7.75	2.48	4350
PZT-8	300	225	1000	0.004	0.48	7.65	2.5	4580

**Table 2:** Properties of polymer phase materials for selection.

Material Type	Epoxy Resin	PDMS
$\varepsilon_{33}/\varepsilon_{0}$	3–4	2.75
Young’s modulus (kPa)	4.0 × 10^6^	750
Density ρ (Kg/m^3^)	1140	970
Longitudinal wave velocity (m/s)	2400–2900	1000
Acoustic impedance (10^6^ g/(cm^2^ × s))	0.27–0.36	0.097

**Table 3:** Performance tests.

Sample	$f_{r}\;$	$f_{r2}\;$	$K_{t}\;$	***μ***	***ρ***	***Z***	$\varepsilon_{r}\;$
1	157	396	0.67	3084	5604	17.283	1121
2	158	392	0.68	3100	5600	17.360	1120
3	157	395	0.65	3080	5601	17.251	1118
4	156	396	0.67	3080	5602	17.254	1120
5	157	395	0.64	3072	5600	17.203	1118

**Table 4:** The comparison between the experimental value and the experimental value.

Sample	$\Delta f_{r}$ %	$\Delta f_{r2}$ %	$\Delta K_{t}\%$	Δ ***μ%***	Δ ***ρ%***	Δ ***Z%***
1	1.86	1.00	1.47	6.54	1.89	4.75
2	1.25	2.00	0.00	6.06	1.82	5.21
3	1.86	1.25	4.41	6.67	1.84	4.55
4	2.50	1.00	1.47	6.67	1.85	4.57
5	1.86	1.25	5.88	6.91	1.82	4.26

**Table 5:** Test result of doped PDMS + annular transducer.

**Sample**	**−3 dB (kHz)**	**−6 dB (kHz)**	$f_{1}\;\left(kHz\right)$	$f_{u}\;\left(kHz\right)$	**V_p-p_ (V)**	**S_rel_ (dB)**
1#	100	165	60	225	15.40	17.73
2#	98	162	62	224	15.90	18.01
3#	102	167	58	225	15.10	17.56
4#	104	168	58	226	15.80	17.95
5#	100	165	60	225	15.20	17.62

**Table 6:** Test result of epoxy resin + annular transducer.

**Sample**	**−3 dB (kHz)**	**−6 dB(kHz)**	$f_{1}\;\left(kHz\right)$	$f_{u}\;\left(kHz\right)$	**V_p-p_ (V)**	**S_rel_ (dB)**
6#	20	36	142	178	1.04	0.34
7#	26	40	140	180	1.10	0.83
8#	22	38	142	180	1.08	0.67
9#	22	38	142	180	1.08	0.67
10#	20	36	142	178	1.04	0.34

## Data Availability

Not applicable.
